# Supplementary material for: Salmonella multimutants enable efficient identification of SPI-2 effector protein function in gut inflammation and systemic colonization
Source: Nat Commun. 2025 Nov 6;16:9795. doi: 10.1038/s41467-025-64760-w (PMC12592711; doi:10.1038/s41467-025-64760-w)
Supplement: Supplementary file 1 — Supplementary Information [file 41467_2025_64760_MOESM1_ESM.pdf]

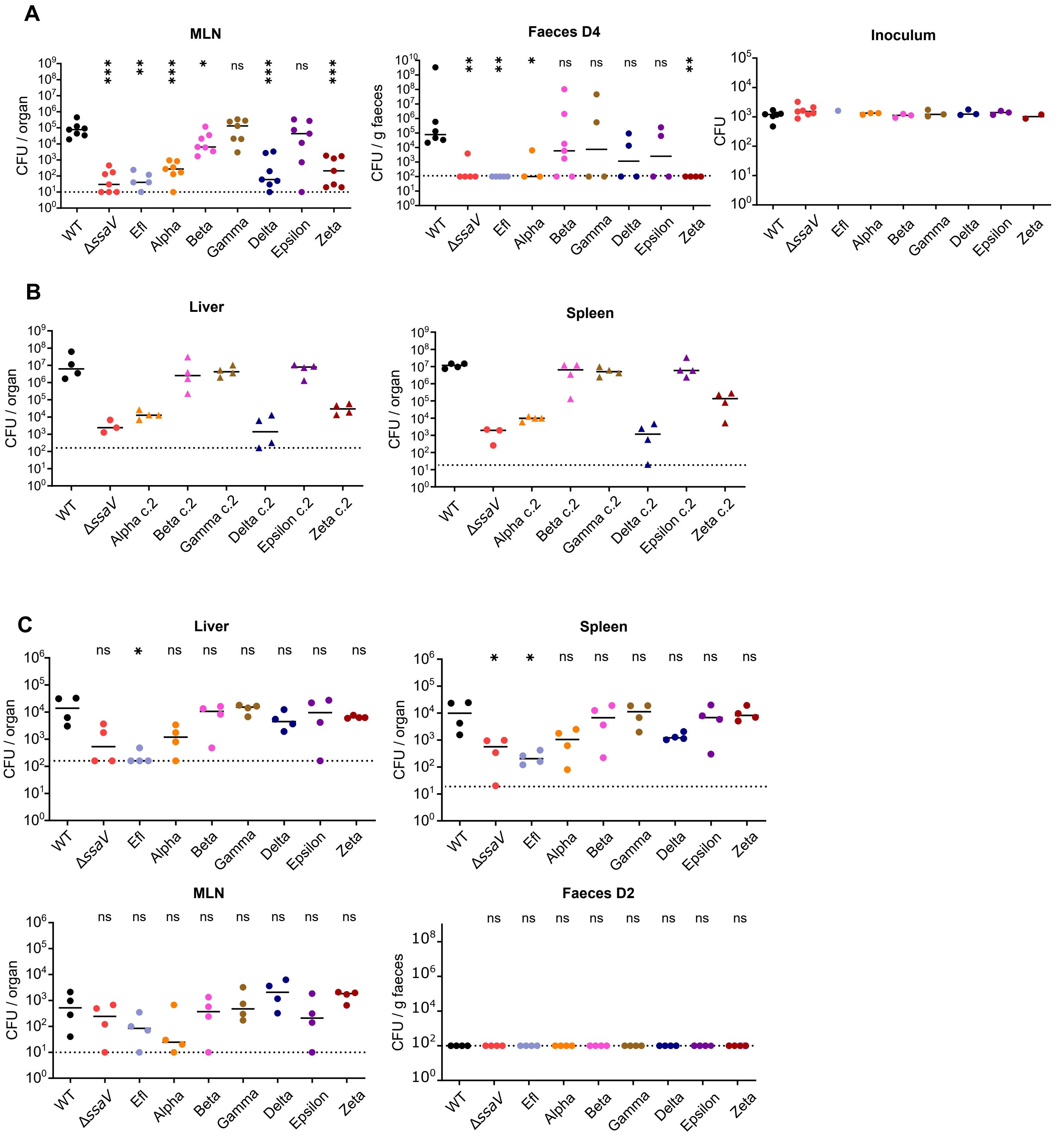
A
MLN
Faeces D4
Inoculum
CFU / organ
CFU / g faeces
CFU
WT
ΔssaV
Efl
Alpha
Beta
Gamma
Delta
Epsilon
Zeta
B
Liver
Spleen
Alpha c.2
Beta c.2
Gamma c.2
Delta c.2
Epsilon c.2
Zeta c.2
C
Liver
Spleen
MLN
Faeces D2

Fig. S1

Supplementary Figure 1. Mulitmutant clones are impaired for systemic infection at various timepoints.

A) Bacterial loads recovered from the mesenteric lymph node (left) and faeces (middle) at day 4 post infection, (*n* = 5-7 mice per group), despite equivalent CFU in the inocula given to mice (right). **B)** CFU for the second independently-constructed clone of each multimutant (triangles), recovered from the liver (left) and spleen (right) at day 4 post infection. **C)** Bacterial loads recovered at day 2 post infection in the liver (upper left), spleen (upper right), mesenteric lymph node (bottom left), and faeces (bottom right). Horizontal bars denote median. Dotted lines denote limit of detection. Statistical differences between WT and indicated groups determined by two-tailed Mann Whitney-U test, ($p \geq 0.05$ not significant (ns), $p<0.05$ (*), $p<0.01$ (**), $p<0.001$ (***). Data pertains to **Fig 2B**.

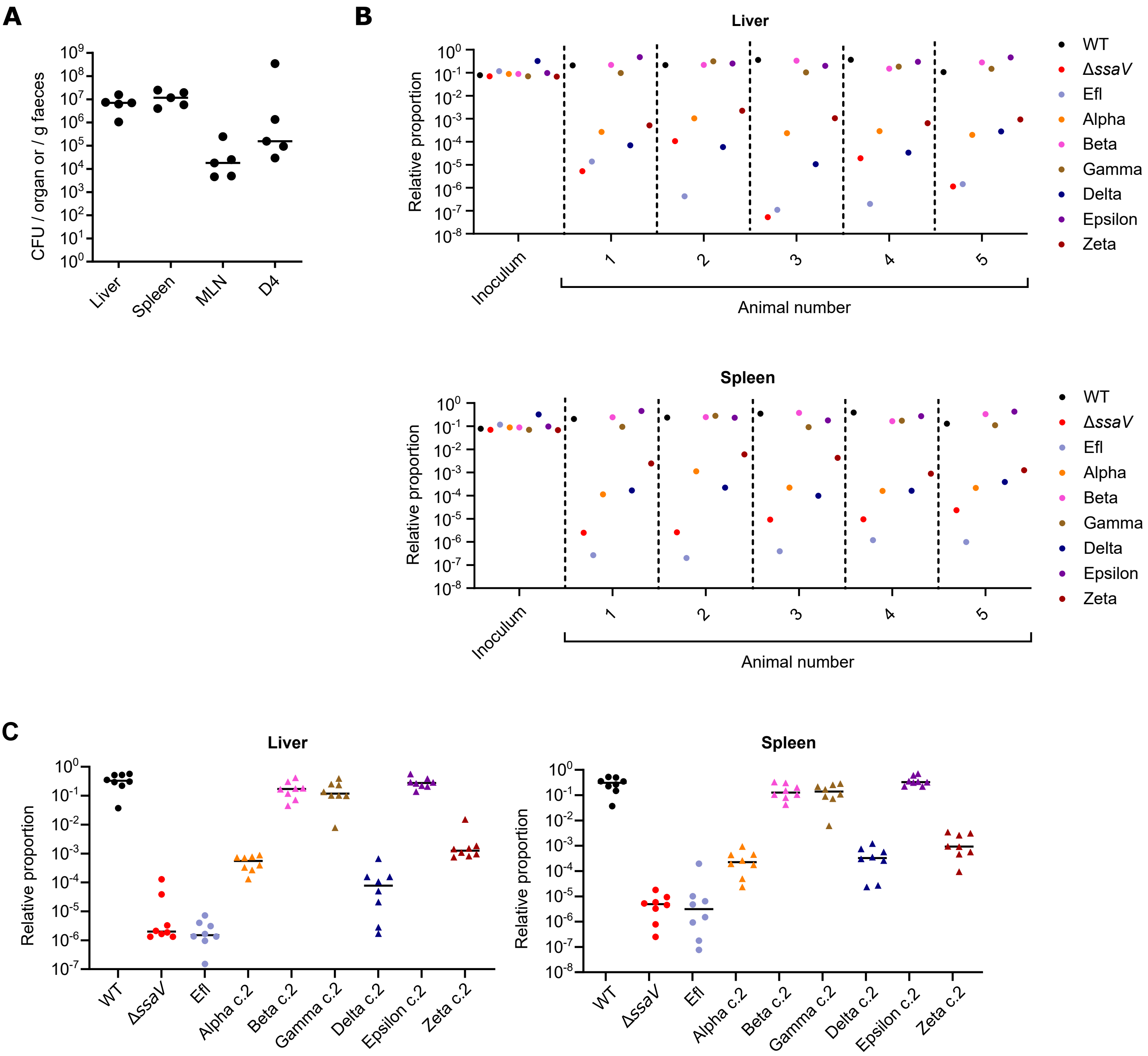
A
CFU / organ or / g faeces
Liver
Spleen
MLN
D4
B
Liver
Spleen
Relative proportion
Inoculum
Animal number
WT
ΔssaV
Efl
Alpha
Beta
Gamma
Delta
Epsilon
Zeta
C
Liver
Spleen
Relative proportion
WT
ΔssaV
Efl
Alpha c.2
Beta c.2
Gamma c.2
Delta c.2
Epsilon c.2
Zeta c.2

Fig. S2

Supplementary Figure 2. Relative fitness of multimutant strains during systemic infection.

A) Bacterial loads at indicated organs and in faeces at day 4 post infection. **B)** Relative proportion of tagged strains recovered from the liver (upper) and spleen (lower) of individual animals, as denoted on the X axis. **C)** Relative proportion of the second independently-constructed clone of each multimutant (triangles) during competitive index infections. Data shows relative proportion of nine strains recovered from the liver (left) and spleen (right) of mice at day 4 post infection. Horizontal bars denote median. Data pertains to **Fig 2D.**

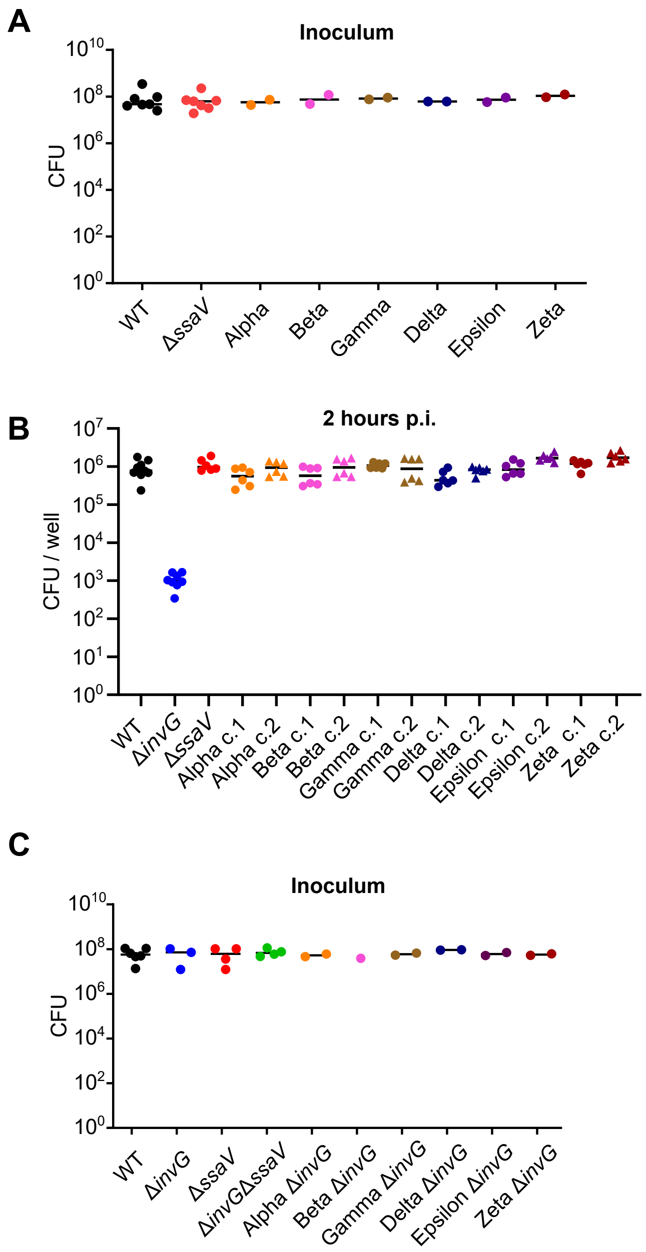
A
Inoculum
CFU
10^10
10^8
10^6
10^4
10^2
10^0
WT
ΔssaV
Alpha
Beta
Gamma
Delta
Epsilon
Zeta
B
2 hours p.i.
CFU / well
10^7
10^6
10^5
10^4
10^3
10^2
10^1
10^0
WT
ΔinvG
ΔssaV
Alpha c.1
Alpha c.2
Beta c.1
Beta c.2
Gamma c.1
Gamma c.2
Delta c.1
Delta c.2
Epsilon c.1
Epsilon c.2
Zeta c.1
Zeta c.2
C
Inoculum
CFU
10^10
10^8
10^6
10^4
10^2
10^0
WT
ΔinvG
ΔssaV
ΔinvGΔssaV
Alpha ΔinvG
Beta ΔinvG
Gamma ΔinvG
Delta ΔinvG
Epsilon ΔinvG
Zeta ΔinvG

Fig. S3

Supplementary Figure 3. Multimutant strains show equivalent CFU in inocula and no invasion defect.

A) CFU for indicated strains determined by selective plating of inocula used for oral infection in **Fig. 3. B)** CFU recovered following *in vitro* infection of HeLa cells with indicated strains at MOI 50. Cells were infected for approximately 2 hours, and CFU enumerated by plating. Circles and triangles denote independently constructed multimutant clones 1 and 2 respectively. **C)** CFU for indicated strains determined by selective plating of inocula used for oral infection in **Fig. 4.** Horizontal bar denotes median.

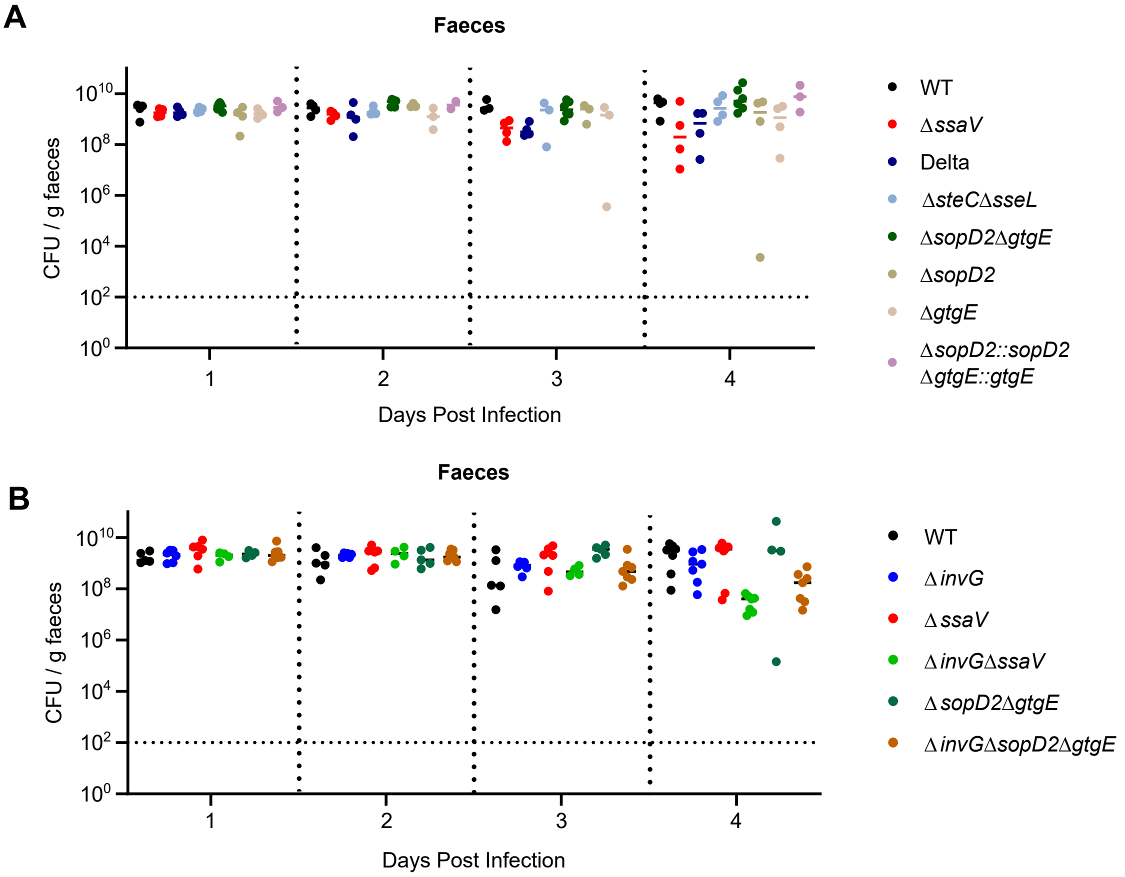
A
Faeces
CFU / g faeces
10^10
10^8
10^6
10^4
10^2
10^0
1
2
3
4
Days Post Infection
WT
ΔssaV
Delta
ΔsteCΔsseL
ΔsopD2ΔgtgE
ΔsopD2
ΔgtgE
ΔsopD2::sopD2
ΔgtgE::gtgE
B
Faeces
CFU / g faeces
10^10
10^8
10^6
10^4
10^2
10^0
1
2
3
4
Days Post Infection
WT
ΔinvG
ΔssaV
ΔinvGΔssaV
ΔsopD2ΔgtgE
ΔinvGΔsopD2ΔgtgE

Fig. S4

Supplementary Figure 4. *Salmonella* carriage in the gut over time during oral infection.

A-B) Bacterial populations in the gut determined by CFU plating of homogenised faecal samples to selective media. Horizontal bar denotes median. Dotted line at 10^2 CFU / g faeces denotes conservative limit of detection. **A)** Data pertains to **Fig. 5A**, (*n* = 3-6 mice per group). **B)** Data pertains to **Fig. 5B**, (*n* = 4-6 mice per group).

Supplementary Table 1. Single mutants used in construction of multimutant strains*

Multimutant	Single mutant	Strain background	Strain ID
S.Tm Alpha	sseFsseG::aphT	SL1344	Z6643, Z6644
S.Tm Alpha	sifA::cat	14028	C4719
S.Tm Alpha	sseJ::aphT	14028	C0955
S.Tm Alpha	pipB2::cat	14028	C4869
S.Tm Alpha	steA::aphT	14028	C0845
S.Tm Beta	sseK1::cat	14028	C4961
S.Tm Beta	sseK1::aphT	14028	C1137
S.Tm Beta	sseK2::cat	14028	C4815
S.Tm Beta	sseK2::aphT	14028	C0991
S.Tm Beta	sseK3::aphT	SL1344	Z5618
S.Tm Beta	gtgA::aphT	14028	C1060
S.Tm Beta	gogA::cat	14028	C5499
S.Tm Beta	pipA::aphT	14028	C1569
S.Tm Gamma	steD::aphT	14028	C1542
S.Tm Gamma	srgE::cat	SL1344	Z6513
S.Tm Gamma	sseI::aphT	14028	C0863
S.Tm Gamma	srfJ::cat	14028	C5374
S.Tm Gamma	steE::aphT	14028	C1356
S.Tm Gamma	gogB::aphT	SL1344	Z6551
S.Tm Delta	steC::aphT	14028	C0867
S.Tm Delta	sseL::cat	SL1344	Z6545
S.Tm Delta	sopD2::aphT	14028	C0861
S.Tm Delta	gtgE::cat	SL1344	Z5685
S.Tm Epsilon	steB::cat	SL1344	Z6525, Z6526
S.Tm Epsilon	cigR::aphT	14028	C1869
S.Tm Epsilon	sspH2::aphT	SL1344	Z8135
S.Tm Epsilon	pipB::cat	SL1344	Z5693
S.Tm Epsilon	slrP::aphT	14028	C0857
S.Tm Epsilon	sifB::cat	14028	C4773
S.Tm Zeta	spvR::aphT	14028	C0780

*Indicated multimutant strains (first column) were constructed by multiple rounds of P22 transduction, using P22 lysates containing single gene deletions (second column). Single mutants were originally constructed in either SL1344 or 14028S background (third column) and are identified with unique strain numbers (fourth column).

Supplementary Table 2. Strains used in this study

Strain name	Strain number	Relevant genotype	Resistance*	Reference
*S.*Tm SL1344	SB300	Wild-type	Sm	(1)
*S.*Tm Δ*invG*	SB161	Δ*invG*	Sm	(2)
*S.*Tm Δ*ssaV*	M2730	Δ*ssaV*	Sm	(3)
*S.*Tm Δ*invG*Δ*ssaV*	M2702	Δ*invG* Δ*ssaV*	Sm	(3)
*S.*Tm Efl	NA170	Δ*spvR* Δ*pipAB* Δ*pipB2* Δ*gtgA* Δ*sifB* Δ*gtgEsseI* Δ*steBsseJ* Δ*pipD* Δ*sseL* Δ*gogBsteE* Δ*sopD2* Δ*slrp* Δ*steA* Δ*steDsseK2* Δ*sspH2* Δ*sseK3* Δ*steC* Δ*cigR* Δ*sseK1* Δ*srfJ* Δ*sseFG* Δ*sifA*	Sm	(4)
*S.*Tm Alpha clone 1	T2978	Δ*sifA* Δ*sseJ* Δ*sseFG* Δ*pipB2* Δ*steA*	Sm	This study
*S.*Tm Alpha clone 2	T2979	Δ*sifA* Δ*sseJ* Δ*sseFG* Δ*pipB2* Δ*steA*	Sm	This study
*S.*Tm Beta clone 1	T2918	Δ*sseK1* Δ*sseK2* Δ*sseK3* Δ*gtgA* Δ*gogA* Δ*pipA*	Sm	This study
*S.*Tm Beta clone 2	T2919	Δ*sseK1* Δ*sseK2* Δ*sseK3* Δ*gtgA* Δ*gogA* Δ*pipA*	Sm	This study
*S.*Tm Gamma clone 1	T2974	Δ*steD* Δ*srgE* Δ*sseI* Δ*srfJ* Δ*steE* Δ*gogB*	Sm	This study
*S.*Tm Gamma clone 2	T2988	Δ*steD* Δ*srgE* Δ*sseI* Δ*srfJ* Δ*steE* Δ*gogB*	Sm	This study
*S.*Tm Delta clone 1	T2982	Δ*sopD2* Δ*gtgE* Δ*steC* Δ*sseL*	Sm	This study
*S.*Tm Delta clone 2	T2984	Δ*sopD2* Δ*gtgE* Δ*steC* Δ*sseL*	Sm	This study
*S.*Tm Epsilon clone 1	T2968	Δ*steB* Δ*cigR* Δ*sspH2* Δ*pipB* Δ*sifB* Δ*slrP*	Sm	This study
*S.*Tm Epsilon clone 2	T2966	Δ*steB* Δ*cigR* Δ*sspH2* Δ*pipB* Δ*sifB* Δ*slrP*	Sm	This study
*S.*Tm Zeta clone 1	T2860	Δ*spvR*	Sm	This study
*S.*Tm Zeta clone 2	T2861	Δ*spvR*	Sm	This study
*S.*Tm WT Tag 1	T3415	WISH 2	Sm, Amp	(5)
*S.*Tm Δ*ssaV* Tag 2	T2949	Δ*ssaV* WISH 7	Sm, Amp	This study
*S.*Tm Efl Tag 3	Z8294	Δ*spvR* Δ*pipAB* Δ*pipB2* Δ*gtgA* Δ*sifB* Δ*gtgEsseI* Δ*steBsseJ* Δ*pipD* Δ*sseL* Δ*gogBsteE* Δ*sopD2* Δ*slrp* Δ*steA* Δ*steDsseK2* Δ*sspH2* Δ*sseK3* Δ*steC* Δ*cigR* Δ*sseK1* Δ*srfJ* Δ*sseFG* Δ*sifA* WISH 3	Sm, Amp	This study
*S.*Tm Alpha clone 1 Tag 4	T7400	Δ*sifA* Δ*sseJ* Δ*sseFG* Δ*pipB2* Δ*steA* WISH 5	Sm, Amp	This study

S.Tm Beta clone 1 Tag 5	T2964	Δ*sseK1* Δ*sseK2* Δ*sseK3* Δ*gtgA* Δ*gogA* Δ*pipA* WISH 49	Sm, Amp	This study
S.Tm Gamma clone 1 Tag 6	T7403	Δ*steD* Δ*srgE* Δ*sseI* Δ*srfJ* Δ*steE* Δ*gogB* WISH 19	Sm, Amp	This study
S.Tm Delta clone 1 Tag 7	T7401	Δ*sopD2* Δ*gtgE* Δ*steC* Δ*sseL* WISH 10	Sm, Amp	This study
S.Tm Epsilon clone 1 Tag 8	T7402	Δ*steB* Δ*cigR* Δ*sspH2* Δ*pipB* Δ*sifB* Δ*slrP* WISH 15	Sm, Amp	This study
S.Tm Zeta clone 1 Tag 9	T2950	Δ*spvR* WISH 16	Sm, Amp	This study
S.Tm Alpha clone 2 Tag 10	T7404	Δ*sifA* Δ*sseJ* Δ*sseFG* Δ*pipB2* Δ*steA* WISH 34	Sm, Amp	This study
S.Tm Beta clone 2 Tag 11	T7406	Δ*sseK1* Δ*sseK2* Δ*sseK3* Δ*gtgA* Δ*gogA* Δ*pipA* WISH 9	Sm, Amp	This study
S.Tm Gamma clone 2 Tag 12	T7408	Δ*steD* Δ*srgE* Δ*sseI* Δ*srfJ* Δ*steE* Δ*gogB* WISH 21	Sm, Amp	This study
S.Tm Delta clone 2 Tag 13	T7410	Δ*sopD2* Δ*gtgE* Δ*steC* Δ*sseL* WISH 40	Sm, Amp	This study
S.Tm Epsilon clone 2 Tag 14	T7412	Δ*steB* Δ*cigR* Δ*sspH2* Δ*pipB* Δ*sifB* Δ*slrP* WISH 51	Sm, Amp	This study
S.Tm Zeta clone 2 Tag 15	T7414	Δ*spvR* WISH 55	Sm, Amp	This study
S.Tm Alpha Δ*invG*	T2990	Δ*sifA* Δ*sseJ* Δ*sseFG* Δ*pipB2* Δ*steA* Δ*invG*	Sm	This study
S.Tm Beta Δ*invG*	T2954	Δ*sseK1* Δ*sseK2* Δ*sseK3* Δ*gtgA* Δ*gogA* Δ*pipA* Δ*invG*	Sm	This study
S.Tm Gamma Δ*invG*	T2992	Δ*steD* Δ*srgE* Δ*sseI* Δ*srfJ* Δ*steE* Δ*gogB* Δ*invG*	Sm	This study
S.Tm Delta Δ*invG*	T2994	Δ*sopD2* Δ*gtgE* Δ*steC* Δ*sseL* Δ*invG*	Sm	This study
S.Tm Epsilon Δ*invG*	T2996	Δ*steB* Δ*cigR* Δ*sspH2* Δ*pipB* Δ*sifB* Δ*slrP* Δ*invG*	Sm	This study
S.Tm Zeta Δ*invG*	T2998	Δ*spvR* Δ*invG*	Sm	This study
S.Tm Δ*sopD2*	Z5675	Δ*sopD2::aphT*	Sm, Kan	This study
S.Tm Δ*gtgE*	Z5685	Δ*gtgE::cat*	Sm, Cm	This study
S.Tm Δ*steC* Δ*sseL*	Z8278	Δ*steC* Δ*sseL*	Sm	This study
S.Tm Δ*sopD2* Δ*gtgE*	T2816	Δ*sopD2::aphT* Δ*gtgE::cat*	Sm, Kan, Cm	This study
S.Tm Δ*sopD2::sopD2* Δ*gtgE::gtgE*	T2856	Δ*sopD2::sopD2* Δ*gtgE::gtgE*	Sm	This study
S.Tm Δ*sopD2* Δ*gtgE* Δ*invG*	T2824	Δ*sopD2::aphT* Δ*gtgE::cat* Δ*invG*	Sm, Kan, Cm	This study
S.Tm Alpha parent 1 clone 1	Z6643	Δ*sseF*Δ*sseG*::*aphT*	Sm, Kan	This study
S.Tm Alpha parent 1 clone 2	Z6644	Δ*sseF*Δ*sseG*::*aphT*	Sm, Kan	This study
S.Tm Alpha parent 2 clone 1	Z6655	Δ*sseF*Δ*sseG*::*aphT* Δ*sifA*::*cat*	Sm, Kan, Cm	This study
S.Tm Alpha parent 2 clone 2	Z6656	Δ*sseF*Δ*sseG*::*aphT* Δ*sifA*::*cat*	Sm, Kan, Cm	This study

*S.*Tm Alpha parent 3 clone 1	Z6681	*ΔsseFΔsseG ΔsifA*	Sm	This study
*S.*Tm Alpha parent 3 clone 2	Z6682	*ΔsseFΔsseG ΔsifA*	Sm	This study
*S.*Tm Alpha parent 4 clone 1	Z6693	*ΔsseFΔsseG ΔsifA ΔsseJ::aphT*	Sm, Kan	This study
*S.*Tm Alpha parent 4 clone 2	Z6694	*ΔsseFΔsseG ΔsifA ΔsseJ::aphT*	Sm, Kan	This study
*S.*Tm Alpha parent 5 clone 1	Z8161	*ΔsseFΔsseG ΔsifA ΔsseJ::aphT ΔpipB2::cat*	Sm, Kan, Cm	This study
*S.*Tm Alpha parent 5 clone 2	Z8162	*ΔsseFΔsseG ΔsifA ΔsseJ::aphT ΔpipB2::cat*	Sm, Kan, Cm	This study
*S.*Tm Alpha parent 6 clone 1	Z8199	*ΔsseFΔsseG ΔsifA ΔsseJ ΔpipB2*	Sm	This study
*S.*Tm Alpha parent 6 clone 2	Z8200	*ΔsseFΔsseG ΔsifA ΔsseJ ΔpipB2*	Sm	This study
*S.*Tm Alpha parent 7 clone 1	T2976	*ΔsseFΔsseG ΔsifA ΔsseJ ΔpipB2 steA::aphT*	Sm, Kan	This study
*S.*Tm Alpha parent 7 clone 2	T2977	*ΔsseFΔsseG ΔsifA ΔsseJ ΔpipB2 steA::aphT*	Sm, Kan	This study
*S.*Tm Beta parent 1 clone 1	Z5608	*ΔsseK2::cat*	Sm, Cm	This study
*S.*Tm Beta parent 1 clone 2	Z5600	*ΔsseK1::cat*	Sm, Cm	This study
*S.*Tm Beta parent 2 clone 1	Z5628	*ΔsseK2::cat ΔsseK3::aphT*	Sm, Kan, Cm	This study
*S.*Tm Beta parent 2 clone 2	Z5624	*ΔsseK1::cat ΔsseK2::aphT*	Sm, Kan, Cm	This study
*S.*Tm Beta parent 3 clone 1	Z5640	*ΔsseK2 ΔsseK3*	Sm	This study
*S.*Tm Beta parent 3 clone 2	Z5636	*ΔsseK1 ΔsseK2*	Sm	This study
*S.*Tm Beta parent 4 clone 1	Z5648	*ΔsseK2 ΔsseK3 ΔsseK1::aphT*	Sm, Kan	This study
*S.*Tm Beta parent 4 clone 2	Z5650	*ΔsseK1 ΔsseK2 ΔsseK3::aphT*	Sm, Kan	This study
*S.*Tm Beta parent 5 clone 1	Z5654	*ΔsseK2 ΔsseK3 ΔsseK1*	Sm	This study
*S.*Tm Beta parent 5 clone 2	Z5656	*ΔsseK1 ΔsseK2 ΔsseK3*	Sm	This study
*S.*Tm Beta parent 6 clone 1	T2900	*ΔsseK2 ΔsseK3 ΔsseK1 gtgA::aphT*	Sm, Kan	This study
*S.*Tm Beta parent 6 clone 2	T2901	*ΔsseK1 ΔsseK2 ΔsseK3 gtgA::aphT*	Sm, Kan	This study
*S.*Tm Beta parent 7 clone 1	T2904	*ΔsseK2 ΔsseK3 ΔsseK1 gtgA::aphT gogA::cat*	Sm, Kan, Cm	This study
*S.*Tm Beta parent 7 clone 2	T2905	*ΔsseK1 ΔsseK2 ΔsseK3 gtgA::aphT gogA::cat*	Sm, Kan, Cm	This study

*S.*Tm Beta parent 8 clone 1	T2914	*ΔsseK2 ΔsseK3 ΔsseK1 ΔgtgA ΔgogA*	Sm	This study
*S.*Tm Beta parent 8 clone 2	T2915	*ΔsseK1 ΔsseK2 ΔsseK3 ΔgtgA ΔgogA*	Sm	This study
*S.*Tm Beta parent 9 clone 1	T2916	*ΔsseK2 ΔsseK3 ΔsseK1 ΔgtgA ΔgogA pipA::aphT*	Sm, Kan	This study
*S.*Tm Beta parent 9 clone 2	T2917	*ΔsseK1 ΔsseK2 ΔsseK3 ΔgtgA ΔgogA pipA::aphT*	Sm, Kan	This study
*S.*Tm Gamma parent 1 clone 1	Z6539	*ΔsteD::aphT*	Sm, Kan	This study
*S.*Tm Gamma parent 1 clone 2	Z6540	*ΔsteD::aphT*	Sm, Kan	This study
*S.*Tm Gamma parent 2 clone 1	Z8117	*ΔsteD::aphT ΔsrgE::cat*	Sm, Kan, Cm	This study
*S.*Tm Gamma parent 2 clone 2	Z8118	*ΔsteD::aphT ΔsrgE::cat*	Sm, Kan, Cm	This study
*S.*Tm Gamma parent 3 clone 1	Z8193	*ΔsteD ΔsrgE*	Sm	This study
*S.*Tm Gamma parent 3 clone 2	Z8194	*ΔsteD ΔsrgE*	Sm	This study
*S.*Tm Gamma parent 4 clone 1	Z8211	*ΔsteD ΔsrgE ΔsseI::aphT*	Sm, Kan	This study
*S.*Tm Gamma parent 4 clone 2	Z8212	*ΔsteD ΔsrgE ΔsseI::aphT*	Sm, Kan	This study
*S.*Tm Gamma parent 5 clone 1	Z8215	*ΔsteD ΔsrgE ΔsseI::aphT ΔsrfJ::cat*	Sm, Kan, Cm	This study
*S.*Tm Gamma parent 5 clone 2	Z8216	*ΔsteD ΔsrgE ΔsseI::aphT ΔsrfJ::cat*	Sm, Kan, Cm	This study
*S.*Tm Gamma parent 6 clone 1	Z8221	*ΔsteD ΔsrgE ΔsseI ΔsrfJ*	Sm	This study
*S.*Tm Gamma parent 6 clone 2	Z8222	*ΔsteD ΔsrgE ΔsseI ΔsrfJ*	Sm	This study
*S.*Tm Gamma parent 7 clone 1	Z8227	*ΔsteD ΔsrgE ΔsseI ΔsrfJ ΔsteE::aphT*	Sm, Kan	This study
*S.*Tm Gamma parent 7 clone 2	Z8228	*ΔsteD ΔsrgE ΔsseI ΔsrfJ ΔsteE::aphT*	Sm, Kan	This study
*S.*Tm Gamma parent 8 clone 1	T2902	*ΔsteD ΔsrgE ΔsseI ΔsrfJ ΔsteE*	Sm	This study
*S.*Tm Gamma parent 8 clone 2	T2903	*ΔsteD ΔsrgE ΔsseI ΔsrfJ ΔsteE*	Sm	This study
*S.*Tm Gamma parent 9 clone 1	T2972	*ΔsteD ΔsrgE ΔsseI ΔsrfJ ΔsteE gogB::aphT*	Sm, Kan	This study
*S.*Tm Gamma parent 9 clone 2	T2986	*ΔsteD ΔsrgE ΔsseI ΔsrfJ ΔsteE gogB::aphT*	Sm, Kan	This study
*S.*Tm Delta parent 1 clone 1	Z6535	*ΔsteC::aphT*	Sm, Kan	This study
*S.*Tm Delta parent 1 clone 2	Z6536	*ΔsteC::aphT*	Sm, Kan	This study
*S.*Tm Delta parent 2 clone 1	Z8270	*ΔsteC::aphT ΔsseL::cat*	Sm, Kan, Cm	This study
*S.*Tm Delta parent 2 clone 2	Z8271	*ΔsteC::aphT ΔsseL::cat*	Sm, Kan, Cm	This study

*S.*Tm Delta parent 3 clone 1	Z8278	*ΔsteC ΔsseL*	Sm	This study
*S.*Tm Delta parent 3 clone 2	Z8279	*ΔsteC ΔsseL*	Sm	This study
*S.*Tm Delta parent 4 clone 1	Z8286	*ΔsteC ΔsseL sopD2::aphT*	Sm, Kan	This study
*S.*Tm Delta parent 4 clone 2	Z8289	*ΔsteC ΔsseL sopD2::aphT*	Sm, Kan	This study
*S.*Tm Delta parent 5 clone 1	T2804	*ΔsteC ΔsseL sopD2::aphT gtgE::cat*	Sm, Kan, Cm	This study
*S.*Tm Delta parent 5 clone 2	T2805	*ΔsteC ΔsseL sopD2::aphT gtgE::cat*	Sm, Kan, Cm	This study
*S.*Tm Epsilon parent 1 clone 1	Z6525	*ΔsteB::cat*	Sm, Kan	This study
*S.*Tm Epsilon parent 1 clone 2	Z6526	*ΔsteB::cat*	Sm, Kan	This study
*S.*Tm Epsilon parent 2 clone 1	Z8115	*ΔsteB::cat ΔcigR::aphT*	Sm, Kan, Cm	This study
*S.*Tm Epsilon parent 2 clone 2	Z8116	*ΔsteB::cat ΔcigR::aphT*	Sm, Kan, Cm	This study
*S.*Tm Epsilon parent 3 clone 1	Z8191	*ΔsteB ΔcigR*	Sm	This study
*S.*Tm Epsilon parent 3 clone 2	Z8192	*ΔsteB ΔcigR*	Sm	This study
*S.*Tm Epsilon parent 4 clone 1	Z8209	*ΔsteB ΔcigR ΔsspH2::aphT*	Sm, Kan	This study
*S.*Tm Epsilon parent 4 clone 2	Z8210	*ΔsteB ΔcigR ΔsspH2::aphT*	Sm, Kan	This study
*S.*Tm Epsilon parent 5 clone 1	Z8219	*ΔsteB ΔcigR ΔsspH2::aphT pipB::cat*	Sm, Kan, Cm	This study
*S.*Tm Epsilon parent 5 clone 2	Z8220	*ΔsteB ΔcigR ΔsspH2::aphT pipB::cat*	Sm, Kan, Cm	This study
*S.*Tm Epsilon parent 6 clone 1	Z8223	*ΔsteB ΔcigR ΔsspH2 ΔpipB*	Sm	This study
*S.*Tm Epsilon parent 6 clone 2	Z8224	*ΔsteB ΔcigR ΔsspH2 ΔpipB*	Sm	This study
*S.*Tm Epsilon parent 7 clone 1	T2924	*ΔsteB ΔcigR ΔsspH2 ΔpipB slrP::aphT*	Sm, Kan	This study
*S.*Tm Epsilon parent 7 clone 2	T2925	*ΔsteB ΔcigR ΔsspH2 ΔpipB slrP::aphT*	Sm, Kan	This study
*S.*Tm Epsilon parent 8 clone 1	T2935	*ΔsteB ΔcigR ΔsspH2 ΔpipB slrP::aphT sifB::cat*	Sm, Kan, Cm	This study
*S.*Tm Epsilon parent 8 clone 2	T2936	*ΔsteB ΔcigR ΔsspH2 ΔpipB slrP::aphT sifB::cat*	Sm, Kan, Cm	This study
*S.*Tm Zeta parent 1 clone 1	Z8264	*ΔspvR::aphT*	Sm, Kan	This study
*S.*Tm Zeta parent 1 clone 2	Z8265	*ΔspvR::aphT*	Sm, Kan	This study
*S.*Tm WISH 2	T3172	Wild-type WISH 2	Sm, Amp	(5)
*S.*Tm WISH 3	T3174	Wild-type WISH 3	Sm, Amp	
*S.*Tm WISH 5	T3178	Wild-type WISH 5	Sm, Amp	
*S.*Tm WISH 7	T3182	Wild-type WISH 7	Sm, Amp	

S.Tm WISH 9	T3287	Wild-type WISH 9	Sm, Amp	
S.Tm WISH 10	T3289	Wild-type WISH 10	Sm, Amp	
S.Tm WISH 15	T3204	Wild-type WISH 15	Sm, Amp	
S.Tm WISH 16	T3295	Wild-type WISH 16	Sm, Amp	
S.Tm WISH 19	T3206	Wild-type WISH 19	Sm, Amp	
S.Tm WISH 21	T3209	Wild-type WISH 21	Sm, Amp	
S.Tm WISH 34	T3299	Wild-type WISH 34	Sm, Amp	
S.Tm WISH 40	T3405	Wild-type WISH 40	Sm, Amp	
S.Tm WISH 49	T3186	Wild-type WISH 49	Sm, Amp	
S.Tm WISH 51	T3189	Wild-type WISH 51	Sm, Amp	
S.Tm WISH 55	T3192	Wild-type WISH 55	Sm, Amp	
14028S *ΔsifA::cat*	C4719	*ΔsifA::cat*	Sm, Cm	(6)
14028S *ΔsseJ::aphT*	C0955	*ΔsseJ::aphT*	Sm, Kan	(6)
14028S *ΔpipB2::cat*	C4869	*ΔpipB2::cat*	Sm, Cm	(6)
14028S *ΔsteA::aphT*	C0845	*ΔsteA::aphT*	Sm, Kan	(6)
14028S *ΔsseK2::cat*	C4815	*ΔsseK2::cat*	Sm, Cm	(6)
14028S *ΔsseK1::cat*	C4961	*ΔsseK1::cat*	Sm, Cm	(6)
SL1344 *ΔsseK3::aphT*	Z5618	*ΔsseK3::aphT*	Sm, Kan	This study
14028S *ΔsseK2::aphT*	C0991	*ΔsseK2::aphT*	Sm, Kan	(6)
14028S *ΔsseK1::aphT*	C1137	*ΔsseK1::aphT*	Sm, Kan	(6)
14028S *ΔgtgA::aphT*	C1060	*ΔgtgA::aphT*	Sm, Kan	(6)
14028S *ΔgogA::cat*	C5499	*ΔgogA::cat*	Sm, Cm	(6)
14028S *ΔpipA::aphT*	C1569	*ΔpipA::aphT*	Sm, Kan	(6)
14028S *ΔsteD::aphT*	C1542	*ΔsteD::aphT*	Sm, Kan	(6)
SL1344 *ΔsrgE::cat*	Z6513	*ΔsrgE::cat*	Sm, Cm	This study
14028S *ΔsseI::aphT*	C0863	*ΔsseI::aphT*	Sm, Kan	(6)
14028S *ΔsrfJ::cat*	C5374	*ΔsrfJ::cat*	Sm, Cm	(6)
14028S *ΔsteE::aphT*	C1356	*ΔsteE::aphT*	Sm, Kan	(6)
SL1344 *ΔgogB::aphT*	Z6551	*ΔgogB::aphT*	Sm, Kan	This study
14028S *ΔsteC::aphT*	C0867	*ΔsteC::aphT*	Sm, Kan	(6)
SL1344 *ΔsseL::cat*	Z6545	*ΔsseL::cat*	Sm, Cm	This study
14028S *ΔsopD2::aphT*	C0861	*ΔsopD2::aphT*	Sm, Kan	(6)
SL1344 *ΔgtgE::cat*	Z5685	*ΔgtgE::cat*	Sm, Cm	This study
14028S *ΔcigR::aphT*	C1869	*ΔcigR::aphT*	Sm, Kan	
SL1344 *ΔsspH2::aphT*	Z8135	*ΔsspH2::aphT*	Sm, Kan	This study
SL1344 *ΔpipB::cat*	Z5693	*ΔpipB::cat*	Sm, Cm	This study
14028S *ΔslrP::aphT*	C0857	*ΔslrP::aphT*	Sm, Kan	(6)
14028S *ΔsifB::cat*	C4773	*ΔsifB::cat*	Sm, Cm	(6)
14028S *ΔspvR::aphT*	C0780	*ΔspvR::aphT*	Sm, Kan	(6)

***Resistances:** Sm = 50 µg/ml streptomycin, Amp = 50 µg/ml ampicillin, Cm = 15 µg/ml chloramphenicol, Kan = 50 µg/ml kanamycin.

Supplementary Table 3. Primers used in this study

Primer name	Sequence	Source	Purpose
slrP_FW	GACGACTGTGACCTCTTATTTAAA	This study	Genotyping of slrP deletion
slrP_RV	AAAAAGCGCTACAGGCGTTGG	This study	Genotyping of slrP deletion
sopD2_FW	TTTCTAAACCCAGGCTGATTCAA	This study	Genotyping of sopD2 deletion
sopD2_RV	CCATGTAATGGGTTTGACTGAAA	This study	Genotyping of sopD2 deletion
gtgA_FW	TAGGCAATGAGTCCGGCCA	This study	Genotyping of gtgA deletion
gtgA_RV	CCTTGGCAGGGCTCGCT	This study	Genotyping of gtgA deletion
sseI_FW	TATTGTGAAATTAAGACCAGGAAGA	This study	Genotyping of sseI deletion
sseI_RV	GATGTTGTTGTCGATCTCCAC	This study	Genotyping of sseI deletion
gtgE_FW	ATGCGACAATACAATAAAAACATATCA	This study	Genotyping of gtgE deletion
gtgE_RV	AGCTTCCCCGTAGGAAATTGA	This study	Genotyping of gtgE deletion
pipA_FW	GTTGGCTTTGTCTGAATCATAGC	This study	Genotyping of pipA deletion
pipA_RV	GCCCCTTTGTTTTTTTAGGCG	This study	Genotyping of pipA deletion
pipB_FW	CAAAGCTCTAAATACAAAAATCACC	This study	Genotyping of pipB deletion
pipB_RV	TGAAACTTAGGGGCGGGGTT	This study	Genotyping of pipB deletion
sifA_FW	GCGCCCGCAGTTGAGATAAA	This study	Genotyping of sifA deletion
sifA_RV	GCCTGGCAAGAGGTTACTCA	This study	Genotyping of sifA deletion
sseF_FW	CGGATGCCTCATGGAGTGA	This study	Genotyping of sseF deletion
sseG_RV	CATCGTAAGGATACTGGCAACA	This study	Genotyping of sseG deletion
srgE_FW	ATGAGTTATTGACCACTGAATTTTCT	This study	Genotyping of srgE deletion
srgE_RV	GAGTAACTTTACGACAATTGCTTC	This study	Genotyping of srgE deletion
steA_FW	CTGAAAATGTATGCCTTTGAGCAA	This study	Genotyping of steA deletion
steA_RV	TTCTGAGAATCTCTTTGCGACAC	This study	Genotyping of steA deletion
sifB_FW	AAAGCAAAAATCAGGTGTTTCACC	This study	Genotyping of sifB deletion
sifB_RV	TTCGTTCCATAGTAAATCCATTATTC	This study	Genotyping of sifB deletion
steB_FW	CTTAGTCAATGTGGACAAAAAATCAAA	This study	Genotyping of steB deletion
steB_RV	ACGGCAGAACTTCCCATAGC	This study	Genotyping of steB deletion
sseJ_FW	AAGAAGCGTAATTCCATATACACC	This study	Genotyping of sseJ deletion
sseJ_RV	CAATCGGCAGCAAAGATAGCAT	This study	Genotyping of sseJ deletion

steC_FW	CAAACTGGCAAATCAAAGAGTCT	This study	Genotyping of steC deletion
steC_RV	TTGCATCTCCGCTACAGGCT	This study	Genotyping of steC deletion
sseK3_FW	TTAAGCCCCCCCTAACCAAGTAAAAACTATCGTTTCAGAT	This study	Genotyping of sseK3 deletion
sseK3_RV	TTCACCACGGCACGCAGGTCATCCAATTTAATGGAGGTAC	This study	Genotyping of sseK3 deletion
sseK2_FW	GTCGGACTCAGGACTTAGCATTGTGACGTTAACGTTTAAA	This study	Genotyping of sseK2 deletion
sseK2_RV	TGAAAGTTCTGTAGAGAAACTTGAATGTGAAATTGAGGTA	This study	Genotyping of sseK2 deletion
steD_FW	CCTATTTAGATGATGGCTTAGCG	This study	Genotyping of steD deletion
steD_RV	CTATATAAGTCATAAGCCTCTGGT	This study	Genotyping of steD deletion
sspH2_FW	TCTGCACCTTCTGAAGCCC	This study	Genotyping of sspH2 deletion
sspH2_RV	GTCATCCGGATATTTCACCTGT	This study	Genotyping of sspH2 deletion
sseL_FW	GCAATATCTCTTGTATCGACGC	This study	Genotyping of sseL deletion
sseL_RV	GACAGCAGGTTGGCGATGT	This study	Genotyping of sseL deletion
gogB_FW	TAGGTTCTAAATCTTGCCTGAATG	This study	Genotyping of gogB deletion
gogB_RV	AAGTTGGCATGTAGTCTAGAGTTA	This study	Genotyping of gogB deletion
steE_FW	TCTTGTTGTGATGAGATTCGTATATA	This study	Genotyping of steE deletion
steE_RV	AAATCACACAATCCGGACTGAG	This study	Genotyping of steE deletion
gogA_FW	GCTTTTAGCTTAATTGATTGCGTG	This study	Genotyping of gogA deletion
gogA_RV	ATTCCATTTGAGGCTGCCATTC	This study	Genotyping of gogA deletion
pipB2_FW	TTATTATGTAACCAGACGTAAAGGG	This study	Genotyping of pipB2 deletion
pipB2_RV	TTTTACCGTCGCATACTCCTGT	This study	Genotyping of pipB2 deletion
cigR_FW	ATAAGCTGCTGTTGGCGAGC	This study	Genotyping of cigR deletion
cigR_RV	CGTAGCGAGTCAAACCTCAC	This study	Genotyping of cigR deletion
sseK1_FW	CTGGCAGGGTATTTATGTATCCTCCGGTTAATGCTTAGTT	This study	Genotyping of sseK1 deletion
sseK1_RV	AATGCCGTATATCTCCGTTCTGAACAGCACTGCGATTTTA	This study	Genotyping of sseK1 deletion
srfJ_FW	GACTGGAAACAGCGCTTTATTGATGCC	This study	Genotyping of srfJ deletion
srfJ_RV	GTCGCTTCATTAAATCCCAGCT	This study	Genotyping of srfJ deletion
spvR_FW	CATAATCCTATCCAGTAACCCC	This study	Genotyping of spvB deletion
spvR_RV	GGTGAACTACCGCTATGGAG	This study	Genotyping of spvB deletion
pipB_red_FW	CCTATAAGGAGTCGGCTCACTTCCATAAGAAGGAATCAAAATATGAATATCCTCCTTAGTTCC	This study	Lambda red replacement of pipB

pipB_red_RV	TGTTTGAATACTTCTTGTTTATAAAATCCCTTTATCTCGATGTGTAGGCTGGAGCTGCTTC	This study	Lambda red replacement of pipB
srgE_red_FW	ACTACACTGGGAAATCGTTGCGTGGTGGTTCCGGAGATAGATATGAATATCCTCCTTAGTTCC	This study	Lambda red replacement of srgE
srgE_red_RV	AATGCCAGACTTCCGCTACCAGACGGTATACACAGTATTATGTGTAGGCTGGAGCTGCTTC	This study	Lambda red replacement of srgE
sseJ_red_FW	TTATTTGCTAAAGCGTGTTTAATAAAGTAAGGAGGACACTATATGAATATCCTCCTTAGTTCC	This study	Lambda red replacement of sseJ
sseJ_red_RV	AGCTGTGTTTTGCTCAAGGCGTACCGCAGCCGATGGAACTTGTGTAGGCTGGAGCTGCTTC	This study	Lambda red replacement of sseJ
gtgA_red_FW	AATGTTAATTCCATGTAATAAAAAGGATGTGTAACTCATCATATGAATATCCTCCTTAGTTCC	This study	Lambda red replacement of gtgA
gtgA_red_RV	GTGTTGTAGCATCGTGGGATTTTGCATTTTTTGATGAGTGTGTGTAGGCTGGAGCTGCTTC	This study	Lambda red replacement of gtgA
gtgE_red_FW	TATAATTACATTAACAAAATTACTATTCGGCGAGTATATTATATGAATATCCTCCTTAGTTCC	This study	Lambda red replacement of gtgE
gtgE_red_RV	AATTATCTTGGTAAAGGTTAACTATCATAAAATGGTACACTGTGTAGGCTGGAGCTGCTTC	This study	Lambda red replacement of gtgE
sseL_red_FW	ATTGAGCATACCGCAATTTCACAGCTTATATACAGAAGAGATATGAATATCCTCCTTAGTTCC	This study	Lambda red replacement of sseL
sseL_red_RV	AGGATAAGAGCCTAATGGGATAGGCTCTAAGTACTCACCATGTGTAGGCTGGAGCTGCTTC	This study	Lambda red replacement of sseL
gogB_red_FW	ATTGAAAAAGCGCATGAAAATAGGATTCCAACCAGCCATAATATGAATATCCTCCTTAGTTCC	This study	Lambda red replacement of gogB
gogB_red_RV	GCTCTATATATAAATATATTAATTGCATATTTTTTTAAAGTGTGTAGGCTGGAGCTGCTTC	This study	Lambda red replacement of gogB
gogA_red_FW	AATGTTAATTCCATGTAATAAAAAGGATGTGTAACTCATCATATGAATATCCTCCTTAGTTCC	This study	Lambda red replacement of gogA
gogA_red_RV	GTGTTGTAGCATCGTGGGATTTTGCATTTTTTGATGAGTGTGTGTAGGCTGGAGCTGCTTC	This study	Lambda red replacement of gogA
sseFsseG_red_FW	AATGGTTGATACTCTTATTGCTTAAATAACAGAACGAAATATATGAATATCCTCCTTAGTTCC	This study	Lambda red replacement of sseFsseG
sseFsseG_red_RV	TTTAGAAAGCAATGAACATCCGGTATATACCTGAAAACGATGTGTAGGCTGGAGCTGCTTC	This study	Lambda red replacement of sseFsseG
sspH2_red_FW	CGGACAGATACTATATGTAAATTTATAAAGGTTTTTGTTATATGAATATCCTCCTTAGTTCC	This study	Lambda red replacement of sspH2
sspH2_red_RV	GGAATATCTTTGTCGCACCGCACCTCATTCACCTGGTGCATGTGTAGGCTGGAGCTGCTTC	This study	Lambda red replacement of sspH2
sopD2_red_FW	TTGGATCTTGCTTTCGCGGTAAATAATCAAGGGAGTTATTATATGAATATCCTCCTTAGTTCC	This study	Lambda red replacement of sopD2

sopD2_red_RV	AAAAAAGGCTCCATATCAGTGGGGCCTTTTTAATGACTTTTGTGTAGGCTGGAGCTGCTTC	This study	Lambda red replacement of sopD2
steA_red_FW	GACATATAAAGCTATTGAGCAAAATTTGAAGGAGTAGGATATATGAATATCCTCCTTAGTTCC	This study	Lambda red replacement of steA
steA_red_RV	AGTCTGATTTCTAACAAAACTGGCTAAACATAAACGCTTTTGTGTAGGCTGGAGCTGCTTC	This study	Lambda red replacement of steA
steB_red_FW	TCATTATTGTTAGTTTGAAATCAATCTCAGGTAATAATCCATATGAATATCCTCCTTAGTTCC	This study	Lambda red replacement of steB
steB_red_RV	CTGTGGAATAGCAATGCCGGGAAGGACATGGCATGACACTTGTGTAGGCTGGAGCTGCTTC	This study	Lambda red replacement of steB
steC_red_FW	TTGCATGTGTATTATAATAAATTTTCAGAGGATGAGACATATATGAATATCCTCCTTAGTTCC	This study	Lambda red replacement of steC
steC_red_RV	TGTGCCCCCGGCGATTCGCAGAAAAGAACGGAACTAAATGTGTGTAGGCTGGAGCTGCTTC	This study	Lambda red replacement of steC
sifB_red_FW	CCAGTAATGAAGTATCATATAATCACTTGTGGTCTACATTATATGAATATCCTCCTTAGTTCC	This study	Lambda red replacement of sifB
sifB_red_RV	ATTGCCAGGGGATTGTAAATCCATACTATTTATGGTGTGATGTGTAGGCTGGAGCTGCTTC	This study	Lambda red replacement of sifB
slrP_red_FW	TCTGTTACTTTAGGTTACGTTCAGATCAGGTAGGGAAAATATATGAATATCCTCCTTAGTTCC	This study	Lambda red replacement of slrP
slrP_red_RV	GTAAACAGGCTCTCTCCCTCTTCTGATAAACTGCGTTCAGATATGAATATCCTCCTTAGTTCC	This study	Lambda red replacement of slrP
BamHI_sopD2_FW	ATTTGGATCCACAGGCGCGAAACCAGTC	This study	Complementation insert for sopD2 with restriction sites
NotI_sopD2_RV	ATTTGCGGCCGCATCAAAGGCGATGTTCTGAACTT	This study	Complementation insert for sopD2 with restriction sites
BamHI_gtgE_FW	ATTTGGATCCTTCGGCATCGAGGTCAAAGG	This study	Complementation insert for gtgE with restriction sites
NotI_gtgE_RV	ATTTGCGGCCGCGGGACAGTCATCCGTTTTTAAC	This study	Complementation insert for gtgE with restriction sites
WISH FW	TATGAGGAGAGTAGGAGGCAATGG	(7)	RT-qPCR amplification of all WISH tags (common forward primer)
WISH 2 RV	TCTCTTCTGGGTATGACGGTATCC	(7)	RT-qPCR amplification of Tag 1

WISH 7 RV	TCATCCCATCTAAATCGCCAAAGG	(7)	RT-qPCR amplification of Tag 2
WISH 3 RV	ACGGCATCCCATTATTCCTTTACC	(7)	RT-qPCR amplification of Tag 3
WISH 5 RV	CCTAATCCTGGGCTTTGTGTATCC	(7)	RT-qPCR amplification of Tag 4
WISH 49 RV	AACCATCCGTATCTCAGTCATTGC	(7)	RT-qPCR amplification of Tag 5
WISH 19 RV	ACCATCAGTTCCAATCGCTTATCC	(7)	RT-qPCR amplification of Tag 6
WISH 10 RV	ATGGGCGTATTGAGGTTGTTATCC	(7)	RT-qPCR amplification of Tag 7
WISH 15 RV	ACGATGGACGAAACTACGATAACG	(7)	RT-qPCR amplification of Tag 8
WISH 16 RV	CAAATGAGAGCCGATAAGCAAACG	(7)	RT-qPCR amplification of Tag 9
WISH 34 RV	GAACACTCTCTTACGACGCATAGG	(7)	RT-qPCR amplification of Tag 10
WISH 9 RV	TCAGACATCACCCTACGACTATCC	(7)	RT-qPCR amplification of Tag 11
WISH 21 RV	ACGGTCTTGATTGGCTATGATTCC	(7)	RT-qPCR amplification of Tag 12
WISH 40 RV	TAAAGCAATGGTGTAGCGGTATCC	(7)	RT-qPCR amplification of Tag 13
WISH 51 RV	ACACACAGGCTACTATGCTTATGC	(7)	RT-qPCR amplification of Tag 14
WISH 55 RV	CTCCCGTTGTCCATTCTCATTAGG	(7)	RT-qPCR amplification of Tag 15

Supplementary References

1. Hoiseth SK, Stocker BA. 1981. Aromatic-dependent Salmonella typhimurium are non-virulent and effective as live vaccines. Nature 291:238-9.
2. Kaniga K, Bossio JC, Galán JE. 1994. The Salmonella typhimurium invasion genes invF and invG encode homologues of the AraC and PulD family of proteins. Mol Microbiol 13:555-68.
3. Periaswamy B, Maier L, Vishwakarma V, Slack E, Kremer M, Andrews-Polymenis HL, McClelland M, Grant AJ, Suar M, Hardt WD. 2012. Live attenuated S. Typhimurium vaccine with improved safety in immuno-compromised mice. PLoS One 7:e45433.
4. Chen D, Burford WB, Pham G, Zhang L, Alto LT, Ertelt JM, Winter MG, Winter SE, Way SS, Alto NM. 2021. Systematic reconstruction of an effector-gene network reveals determinants of Salmonella cellular and tissue tropism. Cell Host & Microbe 29:1531-1544.e9.
5. Schubert C, Nguyen BD, Sichert A, Näpflin N, Sintsova A, Feer L, Näf J, Daniel BBJ, Steiger Y, von Mering C, Sauer U, Hardt W-D. 2025. Monosaccharides drive Salmonella gut colonization in a context-dependent or -independent manner. Nature Communications 16:1735.
6. Porwollik S, Santiviago CA, Cheng P, Long F, Desai P, Fredlund J, Srikumar S, Silva CA, Chu W, Chen X, Canals R, Reynolds MM, Bogomolnaya L, Shields C, Cui P, Guo J, Zheng Y, Endicott-Yazdani T, Yang HJ, Maple A, Ragoza Y, Blondel CJ, Valenzuela C, Andrews-Polymenis H, McClelland M. 2014. Defined single-gene and multi-gene deletion mutant collections in Salmonella enterica sv Typhimurium. PLoS One 9:e99820.
7. Daniel BBJ, Steiger Y, Sintsova A, Field CM, Nguyen BD, Schubert C, Cherrak Y, Sunagawa S, Hardt WD, Vorholt JA. 2024. Assessing microbiome population dynamics using wild-type isogenic standardized hybrid (WISH)-tags. Nat Microbiol 9:1103-1116.
