# Supplementary material for: Salmonella multimutants enable efficient identification of SPI-2 effector protein function in gut inflammation and systemic colonization
Source: Nat Commun. 2025 Nov 6;16:9795. doi: 10.1038/s41467-025-64760-w (PMC12592711; doi:10.1038/s41467-025-64760-w)
Supplement: Supplementary file 2 — Description of Additional Supplementary Files [file 41467_2025_64760_MOESM2_ESM.pdf]

Description of Additional Supplementary Files:

Supplementary Data 1: List of polymorphisms detected via whole genome sequencing. Polymorphism detected in twelve multimutant strains (first column) are predicted to be variations arising either from transduction from 14028S background or arising de novo during strain construction (second column). Determination of polymorphisms is based on evidence from read alignment (RA) or new junction (JC) per the breseq pipeline. The chromosomal location of each polymorphism, the nature of the substitution, and the predicted effect on amino acid composition are described in columns ‘pos’, ‘mutation’, and ‘annotation’ respectively. The affected gene is also listed, with arrows denoting the chromosomal strand bearing this gene (left arrow denotes negative strand, right arrow denotes positive strand). A brief description of the indicated gene is also given.
