# Supplementary material for: Salmonella multimutants enable efficient identification of SPI-2 effector protein function in gut inflammation and systemic colonization
Source: Nat Commun. 2025 Nov 6;16:9795. doi: 10.1038/s41467-025-64760-w (PMC12592711; doi:10.1038/s41467-025-64760-w)
Supplement: Supplementary file 4 — Reporting Summary [file 41467_2025_64760_MOESM4_ESM.pdf]

nature portfolio

Corresponding author(s): Wolf-Dietrich Hardt and Joshua Newson

Last updated by author(s): Jul 28, 2025

Reporting Summary

Nature Portfolio wishes to improve the reproducibility of the work that we publish. This form provides structure for consistency and transparency in reporting. For further information on Nature Portfolio policies, see our Editorial Policies and the Editorial Policy Checklist.

Statistics

For all statistical analyses, confirm that the following items are present in the figure legend, table legend, main text, or Methods section.

n/a	Confirmed	
☐	☒	The exact sample size (*n*) for each experimental group/condition, given as a discrete number and unit of measurement
☐	☒	A statement on whether measurements were taken from distinct samples or whether the same sample was measured repeatedly
☐	☒	The statistical test(s) used AND whether they are one- or two-sided<br>*Only common tests should be described solely by name; describe more complex techniques in the Methods section.*
☒	☐	A description of all covariates tested
☐	☒	A description of any assumptions or corrections, such as tests of normality and adjustment for multiple comparisons
☒	☐	A full description of the statistical parameters including central tendency (e.g. means) or other basic estimates (e.g. regression coefficient) AND variation (e.g. standard deviation) or associated estimates of uncertainty (e.g. confidence intervals)
☐	☒	For null hypothesis testing, the test statistic (e.g. *F*, *t*, *r*) with confidence intervals, effect sizes, degrees of freedom and *P* value noted<br>*Give P values as exact values whenever suitable.*
☒	☐	For Bayesian analysis, information on the choice of priors and Markov chain Monte Carlo settings
☒	☐	For hierarchical and complex designs, identification of the appropriate level for tests and full reporting of outcomes
☒	☐	Estimates of effect sizes (e.g. Cohen's *d*, Pearson's *r*), indicating how they were calculated

Our web collection on statistics for biologists contains articles on many of the points above.

Software and code

Policy information about availability of computer code

Data collection	Data was collected as described in the manuscript.
Data analysis	All data was analysed using open source tools or tools that have been used under appropriate academic licenses. All data was plotted using GraphPad Prism 10. Figures were assembled in Inkscape v1.3.2. Some graphical elements were generated with BioRender. For whole-genome sequencing analysis, library preparation and short-read Illumina sequencing were performed by BMKGENE to confirm deletion of target genes and assess the degree of other polymorphisms in the genome. The resulting raw reads were cleaned by removing adaptor sequences, low-quality-end trimming and removal of low-quality reads using BBTools v 38.18 using the parameters trimq=14, maq=20, maxns=0 and minlength=45. (Bushnell, B. BBMap. Available from: https://sourceforge.net/projects/bbmap/.). The genetic changes in the strains as compared to the reference genome (GCF_000210855.2) were identified using breseq (v. 0.38) run in consensus mode with default parameters.

For manuscripts utilizing custom algorithms or software that are central to the research but not yet described in published literature, software must be made available to editors and reviewers. We strongly encourage code deposition in a community repository (e.g. GitHub). See the Nature Portfolio guidelines for submitting code & software for further information.

Data

Policy information about availability of data

All manuscripts must include a data availability statement. This statement should provide the following information, where applicable:

- Accession codes, unique identifiers, or web links for publicly available datasets
- A description of any restrictions on data availability
- For clinical datasets or third party data, please ensure that the statement adheres to our policy

The data generated in this study are provided as Source Data files. The raw sequencing data generated in this study have been deposited in the European Nucleotide Archive (ENA) under accession number PRJEB83585.

Research involving human participants, their data, or biological material

Policy information about studies with human participants or human data. See also policy information about sex, gender (identity/presentation), and sexual orientation and race, ethnicity and racism.

Reporting on sex and gender	NA
Reporting on race, ethnicity, or other socially relevant groupings	NA
Population characteristics	NA
Recruitment	NA
Ethics oversight	NA

Note that full information on the approval of the study protocol must also be provided in the manuscript.

Field-specific reporting

Please select the one below that is the best fit for your research. If you are not sure, read the appropriate sections before making your selection.

☒ Life sciences ☐ Behavioural & social sciences ☐ Ecological, evolutionary & environmental sciences

For a reference copy of the document with all sections, see nature.com/documents/nr-reporting-summary-flat.pdf

Life sciences study design

All studies must disclose on these points even when the disclosure is negative.

Sample size	For in vitro experiments, at least three biological replicates were used. For in vivo experiments, the number of mice per group ranged from three to eight, as described in figure legends and source data. Sample sizes were determined based on prior data from our group and in agreement with local regulations and animal welfare office requirements.
Data exclusions	No data were excluded from the analysis.
Replication	All the experiments were performed at least twice, with the exception of data described in Fig 2D which is derived from one replicate comprising five individual mice. All attempts at data replication were successful.
Randomization	Mice were randomly allocated to different groups.
Blinding	Blinding was not performed, as investigators needed to determine infection groups based on mouse cage numbers.

Reporting for specific materials, systems and methods

We require information from authors about some types of materials, experimental systems and methods used in many studies. Here, indicate whether each material, system or method listed is relevant to your study. If you are not sure if a list item applies to your research, read the appropriate section before selecting a response.

Materials & experimental systems

n/a	Involved in the study
☒	☐ Antibodies
☐	☒ Eukaryotic cell lines
☒	☐ Palaeontology and archaeology
☐	☒ Animals and other organisms
☒	☐ Clinical data
☒	☐ Dual use research of concern
☒	☐ Plants

Methods

n/a	Involved in the study
☒	☐ ChIP-seq
☒	☐ Flow cytometry
☒	☐ MRI-based neuroimaging

Eukaryotic cell lines

Policy information about cell lines and Sex and Gender in Research

Cell line source(s)	WT HeLa cells (ATCC) were used for experiments described in Fig S3B.
Authentication	Cell lines were not autheticated
Mycoplasma contamination	Cell lines were not tested for mycoplasma contamination
Commonly misidentified lines (See ICLAC register)	*Name any commonly misidentified cell lines used in the study and provide a rationale for their use.*

Animals and other research organisms

Policy information about studies involving animals; ARRIVE guidelines recommended for reporting animal research, and Sex and Gender in Research

Laboratory animals	8- to 12-week old C57BL/6 mice (JAX:000664, The Jackson Laboratory) mice were held under specific pathogen-free (SPF) conditions at the ETH Phenomics Centre (EPIC) at ETH Zürich (light/dark cycle 12:12 h, room temperature 21±1 ˚C, humidity 50±10%).
Wild animals	NA
Reporting on sex	Mice were randomly assigned to experimental groups regardless of their sex.
Field-collected samples	*For laboratory work with field-collected samples, describe all relevant parameters such as housing, maintenance, temperature, photoperiod and end-of-experiment protocol OR state that the study did not involve samples collected from the field.*
Ethics oversight	Protocols used were approved by the Cantonal Veterinary Office of the canton Zurich, Switzerland (Kantonales Veterinäramt ZH licenses 108/2022, 109/2022, 158/2019, 193/2016).

Note that full information on the approval of the study protocol must also be provided in the manuscript.

Plants

Seed stocks	*Report on the source of all seed stocks or other plant material used. If applicable, state the seed stock centre and catalogue number. If plant specimens were collected from the field, describe the collection location, date and sampling procedures.*
Novel plant genotypes	*Describe the methods by which all novel plant genotypes were produced. This includes those generated by transgenic approaches, gene editing, chemical/radiation-based mutagenesis and hybridization. For transgenic lines, describe the transformation method, the number of independent lines analyzed and the generation upon which experiments were performed. For gene-edited lines, describe the editor used, the endogenous sequence targeted for editing, the targeting guide RNA sequence (if applicable) and how the editor was applied.*
Authentication	*Describe any authentication procedures for each seed stock used or novel genotype generated. Describe any experiments used to assess the effect of a mutation and, where applicable, how potential secondary effects (e.g. second site T-DNA insertions, mosiacism, off-target gene editing) were examined.*
